# Incidence and outcomes of surgical pulmonary embolectomy in the UK

**DOI:** 10.1093/bjs/znae003

**Published:** 2024-01-17

**Authors:** Amerikos Argyriou, Hunaid Vohra, Jeremy Chan, Eltayeb Mohamed Ahmed, Cha Rajakaruna, Gianni Davide Angelini, Daniel Paul Fudulu

**Affiliations:** Bristol Heart Institute, Bristol Royal Infirmary, University Hospitals Bristol and Weston NHS Foundation Trust, Bristol, UK; Bristol Heart Institute, Bristol Royal Infirmary, University Hospitals Bristol and Weston NHS Foundation Trust, Bristol, UK; Bristol Heart Institute, Bristol Royal Infirmary, University Hospitals Bristol and Weston NHS Foundation Trust, Bristol, UK; Bristol Heart Institute, Bristol Royal Infirmary, University Hospitals Bristol and Weston NHS Foundation Trust, Bristol, UK; Bristol Heart Institute, Bristol Royal Infirmary, University Hospitals Bristol and Weston NHS Foundation Trust, Bristol, UK; Bristol Heart Institute, Bristol Royal Infirmary, University Hospitals Bristol and Weston NHS Foundation Trust, Bristol, UK; Bristol Heart Institute, Bristol Royal Infirmary, University Hospitals Bristol and Weston NHS Foundation Trust, Bristol, UK

## Abstract

**Background:**

Surgical pulmonary embolectomy is rarely used for the treatment of massive acute pulmonary embolism. The aim of this study was to assess the incidence and outcomes of this operation by undertaking a retrospective analysis of a large national registry in the UK.

**Methods:**

All acute pulmonary embolectomies performed between 1996 and 2018 were captured in the National Institute of Cardiovascular Outcomes Research central database. Trends in the number of operations performed during this interval and reported in-hospital outcomes were analysed retrospectively. Multivariable logistic regression was used to identify independent risk factors for in-hospital death.

**Results:**

All 256 patients treated surgically for acute pulmonary embolism during the study interval were included in the analysis. Median age at presentation was 54 years, 55.9% of the patients were men, 48.0% had class IV heart failure symptoms, and 37.5% had preoperative cardiogenic shock. The median duration of bypass was 73 min, and median cross-clamp time was 19 min. Cardioplegic arrest was used in 53.1% of patients. The median duration of hospital stay was 11 days. The in-hospital mortality rate was 25%, postoperative stroke occurred in 5.4%, postoperative dialysis was required in 16%, and the reoperation rate for bleeding was 7.5%. Risk-adjusted multivariable analysis revealed cardiogenic shock (OR 2.54, 95% c.i. 1.05 to 6.21; *P* = 0.038), preoperative ventilation (OR 5.85, 2.22 to 16.35; *P* < 0.001), and duration of cardiopulmonary bypass exceeding 89 min (OR 7.82, 3.25 to 20.42; *P* < 0.001) as significant independent risk factors for in-hospital death.

**Conclusion:**

Surgical pulmonary embolectomy is rarely performed in the UK, and is associated with significant mortality and morbidity. Preoperative ventilation, cardiogenic shock, and increased duration of bypass were significant predictors of in-hospital death.

## Teaser

Surgical pulmonary embolectomy is carried out to remove pulmonary emboli surgically, and restore flow and cardiac output in patients who are acutely unwell. Surgical pulmonary embolectomy has rarely been carried out in the UK in recent years, with few centres undertaking more than one operation a year. This patient cohort carries a high inpatient mortality rate of 25%, with stroke risk of 5.4%. Independent risk factors for death include cardiogenic shock, requiring ventilation, and long duration of cardiopulmonary bypass.

## Introduction

Pulmonary emboli can lead to devastating cardiac sequelae, with patient presentation ranging drastically from mild dyspnoea to sudden cardiac death^1^. In both Europe and the USA, venous thromboembolism is responsible for over 300 000 deaths per year, and represents the third most common cardiovascular cause of death after myocardial infarction and stroke^2–4^. The incidence of pulmonary embolism (PE) across the developed world has been increasing steadily, and mortality has unfortunately only decreased mildly over the past 20 years^5–8^. Left untreated, large pulmonary emboli are associated with a mortality rate of over 65% and the cause of death is commonly failure of the right cardiac ventricle^9^. The pathomechanism comprises right heart stress and eventual failure owing to both mechanical obstruction and circulatory vasoconstrictive mediator release in response to clot formation^10,11^. The right ventricle is thin-walled and so can only minimally compensate for this sudden increase in afterload, with right ventricular (RV) failure ensuing and subsequent dysfunction of the left ventricle in the form of reduced diastolic filling and a drop in coronary perfusion pressure^10^. The above cascade of events can develop insidiously and suddenly, with 25% of all high-risk PEs presenting with sudden cardiac arrest^12^.

The acute management of PE encompasses early anticoagulation followed by a multidisciplinary approach to guide definitive therapy, if required. Systemic thrombolysis using thrombolytic medicines, catheter-directed therapies such as catheter-directed thrombolysis or thrombectomy, and surgical pulmonary embolectomy (SPE) are all tools available to eradicate the clot burden and ultimately decrease RV strain^13^.

According to the European Society of Cardiology 2019 guidelines^13^ for the management of acute PE, SPE is indicated in patients with high-risk PE in whom thrombolysis has failed or is contraindicated (class I recommendation, level C evidence). Prognostic indicators for high-risk PE are haemodynamic instability, high PE severity index, evidence of RV dysfunction on transthoracic echocardiography or CT pulmonary angiography, and raised levels of cardiac troponin^14^. It is worth noting that a minority of centres have used this operation pre-emptively in more stable patients with intermediate-risk PE, with great results^15–17^. This operation can now be done with or without cardiopulmonary bypass (CPB) and without the need for cardioplegia, depending on institution expertise and facilities available^18^. Furthermore, the operation can negate the use of thrombolysis, which comes with small but significantly increased risks of major bleeding and stroke, as highlighted in two large systematic reviews by Chatterjee *et al*.^19^ and Marti *et al*.^20^.

As there are very limited studies on pulmonary embolectomy, particularly in Europe, the aim of this study was to assess the trends, in-hospital outcomes, and risk factors for patients who underwent SPE in the UK during an interval of 22 years.

## Methods

### Study design and setting

Data collected from the National Adult Cardiac Surgery Audit (NACSA), obtained from the National Institute of Cardiovascular Outcomes Research (NICOR) central cardiac database were analysed retrospectively. The definitions of database variables used for this study are available at https://www.nicor.org.uk/national-cardiac-audit-programme/adult-cardiac-surgery-surgery-audit/. The NACSA registry collects demographic as well as preoperative, perioperative, and postoperative clinical data prospectively for all significant adult cardiac surgical procedures performed in the UK. Its central role is in benchmarking surgical practice. The flow of data from input to analysis has been described previously^21^. The data are entered locally and validated at unit level by database managers before uploading through a web portal to NICOR. Further validation is undertaken according to logical rules, and missing data reports are generated for primary variables (for example, EuroSCORE risk factors, patient identifiers, and outcome data). The data are then forwarded to an academic healthcare informatics department for cleaning. Duplicate records are removed, transcriptional discrepancies recoded, and clinical and temporal conflicts resolved. Missing data are determined during the validation stages of data transfer from individual centres. Missing and conflicting data for in-hospital mortality status are backfilled and validated via record linkage to the Office for National Statistics census database. Missing data were handled by exclusion. The percentage of missing data was as follows for baseline characteristics: 11.12% for BMI, 6.64% for duration of CPB, and 19.14% for aortic cross-clamp time. Rates of missing data for the remaining outcome variables were 3.2% for in-hospital mortality, 11.5% for stroke, 12.7% for need for postoperative dialysis and 4.4% for duration of hospital stay. There were no missing data for other variables included in *Tables 1* and *2*.

**Table 1:** Baseline characteristics of patients undergoing acute surgical pulmonary embolectomy stratified by in-hospital death

	Overall (*n* = 256)	Survivors (*n* = 195)	Non-survivors (*n* = 61)	P*
Age (years), median (i.q.r.)	54 (42–65)	53 (39–64)	58 (47–66)	0.082†
**Sex**				0.229
Male	143 (55.9)	113 (57.9)	30 (49)	
Female	113 (44.1)	82 (42.1)	31 (51)	
BMI (kg/m^2^), median (i.q.r.)	28.2 (25.2–32.4)	27.7 (25.2–31.8)	28 (25.4–33.2)	0.340†
Stroke	21 (8.2)	17 (8.7)	4 (7)	0.591
Creatinine > 200 µmol/l	22 (8.6)	12 (6.2)	10 (16)	0.013
Recent myocadial infarction	8 (3.1)	6 (3.1)	2 (3)	> 0.999
Pulmonary disease	18 (7.0)	14 (7.2)	4 (7)	> 0.999
NYHA class IV heart failure	123 (48.0)	90 (46.2)	33 (54)	0.278
Insulin-dependent diabetes mellitus	9 (3.5)	7 (3.6)	2 (3)	> 0.999
Cardiogenic shock	96 (37.5)	58 (29.7)	38 (62)	< 0.001
Peripheral arterial disease	8 (3.1)	6 (3.1)	2 (3)	> 0.999
Preoperative ventilation	62 (24.2)	33 (16.9)	29 (48)	< 0.001
EuroSCORE II value, median (i.q.r.)	4.0 (1.8–8.7)	3.0 (1.7–6.5)	8.6 (3.5–15.0)	< 0.001†
Duration of cardiopulmonary bypass (min), median (i.q.r.)	73 (45–114)	69 (40–96)	100 (62–142)	< 0.001†
Duration of cross-clamp (min), median (i.q.r.)	19 (0–46)	21 (0–46)	19 (0–41)	0.272†
Cardioplegic arrest	136 (53.1)	105 (53.8)	31 (51)	0.679

Values are *n* (%) unless otherwise indicated. NYHA, New York Heart Association. *Pearson’s χ^2^ test or Fisher’s exact test, except † which used Wilcoxon rank-sum test.

**Table 2:** Unadjusted in-hospital outcomes for patients who underwent surgery for acute pulmonary embolus

	No. of patients* (*n =* 256)
**Death**	61 (23.8)
TIA	3 (1.2)
Stroke	12 (4.7)
Postoperative dialysis	35 (13.7)
Reoperation for bleeding	19 (7.4)
Total duration of hospital stay (days), median (i.q.r.)	11 (6–21)

^*^Values are *n* (%) unless otherwise indicated. TIA, transient ischaemic attack.

The Health Research Authority and Health and Care Research Wales approved the study in 2020 (IRAS project ID 257758), and a waiver for patient consent was obtained. This study was conducted in accordance with the Declaration of Helsinki. There was no patient or public involvement in the design of this retrospective database analysis.

### Patients

Patients who had SPE between 1 January 1997 and 1 January 2018 were identified in the NACSA data set.

### Outcomes

The primary outcomes were in-hospital mortality and assessments of trends in the number of procedures. Secondary outcomes included neurological injury (composite outcome of reversible and permanent neurological deficits), postoperative renal impairment requiring dialysis, re-exploration for bleeding or cardiac tamponade, and duration of hospital stay.

### Statistical analysis

Categorical variables are summarized as counts and percentages, and were compared using Pearson’s χ^2^ test or Fisher’s exact. The Shapiro–Wilks test was used to assess the normality of the distribution of continuous data. The continuous data were distributed non-normally and are summarized as median (i.q.r.), with analysis by Wilcoxon rank-sum test.

For the continuous variables age and duration of CPB, the optimal threshold associated with increased mortality was determined using the Younden metric, and these variables were included in the logistic regression model as binary variables^22^. The cut-off associated with increased mortality was above 58 years for age, and longer than 89 min for CPB time. A logistic regression model was used to assess the effect of age over 58 years, sex, preoperative neurological dysfunction, creatinine level over 200 µmol/l, recent myocardial infarction, pulmonary disease, New York Heart Association class IV symptoms, diabetes on insulin, cardiogenic shock on presentation, peripheral arterial disease, preoperative ventilation, duration of CPB over 89 min, cross-clamp, and cardioplegic arrest on a binary outcome of in-hospital mortality. To tackle issues of overfitting because of the relatively small sample size, stepwise backward regression was used; from the initial model with an Akaike information criterion (AIC) of 178.72, a final model was developed with an AIC of 167.24 that retained four co-variables.

## Results

### Baseline characteristics

Between 1 January 1997 and 1 January 2018, 256 SPEs were carried out for acute PE in the UK among a total of 677 323 cardiac surgical operations recorded in the NACSA database (*Table 1*).

The baseline characteristics for this population are summarized in *Table 1*, stratified by in-hospital mortality. There were significant preoperative differences between the two groups in that the survivors were on average less likely to present with a creatinine level of over 200 mmol/l (6.2 *versus* 16%; *P* = 0.013), less likely to present with cardiogenic shock (29.7 *versus* 62%; *P* < 0.001), less likely to require ventilation (16.9 *versus* 48%; *P* < 0.001), and on average presented with a lower EuroSCORE II value (median 3.0 *versus* 8.6; *P* < 0.001). During surgery, survivors were on CPB for a significantly shorter time on average than those who did not survive the admission (69 *versus* 100 min; *P* < 0.001). A total of 153 patients (*59.8*%) required aortic cross-clamping and cardioplegic arrest, and concomitant procedures included: 10 coronary artery bypass graft operations, 6 atrial septal defect closures, 4 aortic valve replacements, 2 mitral valve replacements, 4 mitral valve repairs, and 2 myxoma operations.

**Fig. 1:**
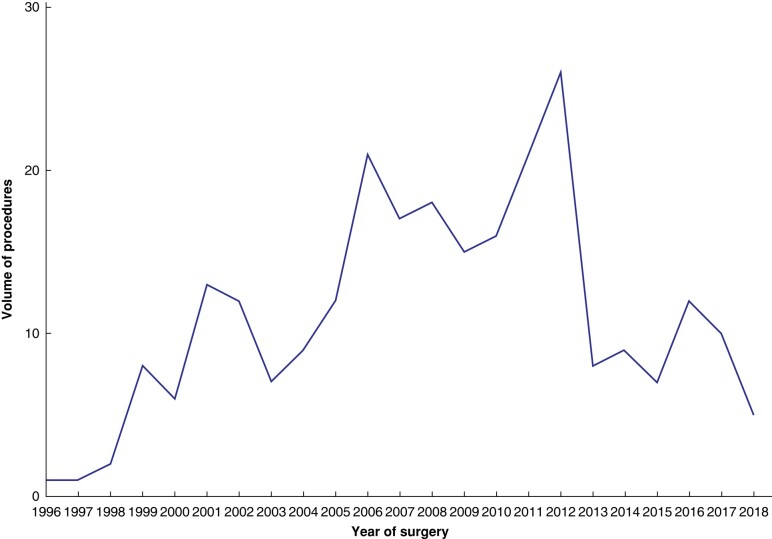
Surgical pulmonary embolectomies carried out between 1 January 1997 and 1 January 2018 (total 256)

The yearly volume of SPE in the UK throughout the study interval was low (*Fig. 1*). Fewer than ten operations per year were carried out in the late 1990s, and this slowly increased to a peak of 24 in 2012 and then dropped to half of this number in more recent years. Of all cardiac operations carried out in the UK between 1997 and 2018, SPE made up only 0.036% recorded in the NACSA database.

**Fig. 2:**
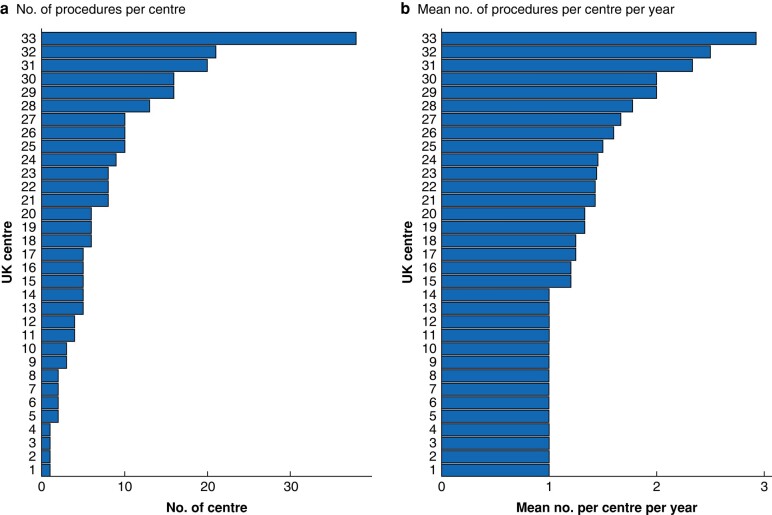
Procedures undertaken in each UK centre (total 256) **a** Number of procedures per centre and **b** mean number of procedures per centre per year.

*Figure 2* shows the total number of procedures per UK centre during the study interval, and the mean number of procedures per centre per year. The total number of procedures per centre ranged between 1 and 38 across 33 centres during the study interval (*Fig. 2*). The figure illustrates that this operation was carried out by nearly all UK cardiac centres during the study interval, but only 6 carried out more than 10 SPEs. The largest number of procedures at a single centre was 38, spanning the 22-year interval.

The mortality rate during admission in this study was 23.8%, the stroke rate was 5.4%, and the median total duration of hospital stay after operation was 11 days (*Table 2*). The results of multivariable stepwise regression analysis of significant variables from our univariate analysis identified independent predictors of short-term mortality for the SPE cohort, including cardiogenic shock (OR 2.54, 95% c.i. 1.05 to 6.21; *P* = 0.038), requirement for preoperative ventilation (OR 5.85, 2.22 to 16.35; *P* < 0.001), and duration of CPB exceeding 89 min (OR 7.82, 3.25 to 20.42; *P* < 0.001) (*Table 3*).

**Table 3:** Predictors of short-term mortality in patients undergoing pulmonary embolectomy selected in the final model after backward stepwise regression

	OR	*P*
Preoperative cardiogenic shock	2.54 (1.05, 6.21)	0.038
Preoperative ventilation	5.85 (2.22, 16.35	< 0.001
Cardiopulmonary bypass exceeding 89 min	7.82 (3.25, 20.42)	< 0.001
Cardioplegic arrest	0.99 (0.97, 1.00)	0.074

Values in parentheses are 95% confidence intervals.

## Discussion

The reported outcomes are derived from the largest national registry outside of the US and Asia, ahead of other European registries to date^23,24^. On average, the UK carried out 12 SPEs a year, but with significant fluctuation in this figure throughout the study interval. There was huge variation in the number of procedures performed across UK centres, ranging from 1 to 38. These findings share predominant similarities with major US-reported outcomes regarding this operation. In the present study, the inpatient mortality rate was 25%, which is similar to major registry findings of that time, with Kilic *et al.*^25^ in 2012 reporting a rate of 27.2%, and Percy *et al.*^26^ 19.8% in an analysis of the US nationwide inpatient sample between 2010 and 2014. Recently reported US registry studies have shown vastly improved mortality rates; in an update of the US nationwide inpatient sample, Alqahtani *et al.*^27^ documented a figure of 14% in a study of 2091 patients who had SPE. At the same time, Kon *et al.*^28^ reported an inpatient mortality rate of 15.9% among patients using the Society of Thoracic Surgeons national database. Interestingly, the inpatient mortality rate was 8% in a smaller case series of 25 critically ill patients requiring ionotropic and ICU support before surgery led by Kadner *et al.*^29^

These more recent papers^27,28^ also reported improved stroke rates of 2.6 and 2.1 respectively, in contrast with the stroke rate of 5.4% in the present study. The authors cannot offer a clear mechanism to explain the increased postoperative stroke rate in this UK cohort. Two hypotheses include a significant proportion of this cohort having a cardiac arrest before SPE, or failed thrombolysis before SPE leading to an increased rate of haemorrhagic stroke. Both of these hypothesized mechanisms of action were represented in patient groups having strokes after SPE in other studies^30,31^, although this is merely a speculative association. A large retrospective study by Lee *et al.*^32^ comparing thrombolysis with SPE, in which each was chosen as first-line therapy, reported that the stroke rate was significantly lower with SPE than with thrombolysis (0.8 *versus* 1.9%). The median duration of hospital stay in the present study was 11 days, which is very similar to the average of 10 days noted in other studies^26,27,33^.

In the present study, cardiogenic shock on presentation, preoperative ventilation, and duration of CPB exceeding 89 min were independent risk factors for death. Cardiogenic shock is a well reported predictor of early death, as noted in all major studies^26,28,33,34^. The median duration of CPB in the total cohort was 73 min in the present series, slightly longer than that reported in other studies (50–70 min)^28,33^. This variable has been associated with decreased survival after SPE in only a single study^28^ in the literature. In the present study, a duration of CPB longer than 89 minutes was associated with increased in-hospital mortality. Prolonged CPB time is a well known predictor of mortality in cardiac surgery. In the context of the present study, the prolonged duration could have been associated with increased complexity of the procedure or inability to wean off CPB owing to RV dysfunction.

Age has been reported as a predictor of death in several large studies, such as Kon *et al.*^28^ (age over 55 years) and Percy *et al*.^26^ (age over 60 years), but this was not replicated here. Other variables cited as being significant predictors of death in major registry studies, but not found to be significant in the present cohort, include BMI, previous myocardial infarction, pulmonary disease, and heart failure^26–28^. Variables that were not available for analysis in the NACSA database, but which have been associated with increased inpatient mortality, include hospital and surgeon case volume, atrial fibrillation, and hypertension at presentation^25,26^. There were no data on ventricular function and the incidence of pulmonary hypertension after operation in the present study. It is worth noting that Keeling *et al.*^15^ reported preserved RV function at a mean of 30 months’ follow-up in their centre’s SPE cohort, whereas Kline *et al.*^35^ noted that a significant proportion of patients treated with thrombolysis after a submassive PE had clinical and echocardiographic evidence of pulmonary hypertension at 6-month follow-up.

Data from the Society for Thoracic Surgery^27^ database indicate that a small proportion of patients in the US SPE cohort undergo thrombolysis (6.2%) and catheter-directed therapies (8.6%) before SPE is attempted. There was no information available in the database used for the present analysis regarding whether this UK cohort had been treated with thrombolysis before SPE, or whether catheter-directed therapies had been attempted. Therefore, it was not possible to assess to what extent these would have influenced outcomes. No data were available on interval from admission to theatre, but other studies^29,36^ have shown that early intervention may be associated with improved outcomes. A US study^27^ reported that over 80% of all SPEs in the USA in recent years were carried out within 48 h of arrival in the emergency department.

Although the present registry study has offered data on the real-world practice of surgical embolectomy in the UK, it is limited by its retrospective nature and the granularity of the data set. No data were available on the indication for SPE, preoperative echocardiography, referral patterns for this cohort, or whether patients underwent thrombolysis before operation. The use of preoperative extracorporeal membrane oxygenation and semielective and surgical embolectomy in high-risk PE has been associated with excellent outcomes^37^; however, such data was not captured in the registry data set used here. Although the authors had data on the number of patients in cardiogenic shock before operation, no information was available on patients with cardiac arrest, which is associated with worse outcomes^38^.

## Data Availability

Requests for data should be directed to the corresponding author. Requests will be assessed for scientific rigour before being granted. Data will be anonymized and transferred securely. A data sharing agreement will be required.

## References

[1] Agnelli G , Becattini C. Acute pulmonary embolism. N Engl J Med 2010;363:266–274 20592294 10.1056/NEJMra0907731

[2] Wendelboe AM , Raskob GE. Global burden of thrombosis: epidemiologic aspects. Circ Res 2016;118:1340–1347 27126645 10.1161/CIRCRESAHA.115.306841

[3] Cohen AT , Agnelli G, Anderson FA, Arcelus JI, Bergqvist D, Brecht JG et al Venous thromboembolism (VTE) in Europe. The number of VTE events and associated morbidity and mortality. Thromb Haemost 2007;98:756–764 17938798 10.1160/TH07-03-0212

[4] Hobohm L , Sebastian T, Valerio L, Mahmoudpour SH, Vatsakis G, Johner F et al Trends in mortality related to pulmonary embolism in the DACH countries. Med Klin Intensivmed Notfmed 2022;117:428–438 34430980 10.1007/s00063-021-00854-9 PMC9452436

[5] Wiener RS , Schwartz LM, Woloshin S. Time trends in pulmonary embolism in the United States: evidence of overdiagnosis. Arch Intern Med 2011;171:831–837 21555660 10.1001/archinternmed.2011.178 PMC3140219

[6] de Miguel-Diez J , Jimenez-Garcia R, Jimenez D, Monreal M, Guijarro R, Otero R et al Trends in hospital admissions for pulmonary embolism in Spain from 2002 to 2011. Eur Respir J 2014;44:942–950 24993910 10.1183/09031936.00194213

[7] Shiraev TP , Omari A, Rushworth RL. Trends in pulmonary embolism morbidity and mortality in Australia. Thromb Res 2013;132:19–25 23725850 10.1016/j.thromres.2013.04.032

[8] Yang Y , Liang L, Zhai Z, He H, Xie W, Peng X et al Pulmonary embolism incidence and fatality trends in Chinese hospitals from 1997 to 2008: a multicenter registration study. PLoS One 2011;6:e26861 22069474 10.1371/journal.pone.0026861 PMC3206059

[9] Jaff MR , McMurtry MS, Archer SL, Cushman M, Goldenberg N, Goldhaber SZ et al Management of massive and submassive pulmonary embolism, iliofemoral deep vein thrombosis, and chronic thromboembolic pulmonary hypertension: a scientific statement from the American Heart Association. Circulation 2011;123:1788–1830 21422387 10.1161/CIR.0b013e318214914f

[10] Turetz M , Sideris AT, Friedman OA, Triphathi N, Horowitz JM. Epidemiology, pathophysiology, and natural history of pulmonary embolism. Semin Intervent Radiol 2018;35:92–98 29872243 10.1055/s-0038-1642036 PMC5986574

[11] Wood KE . Major pulmonary embolism: review of a pathophysiologic approach to the golden hour of hemodynamically significant pulmonary embolism. Chest 2002;121:877–905 11888976 10.1378/chest.121.3.877

[12] Go AS , Mozaffarian D, Roger VL, Benjamin EJ, Berry JD, Blaha MJ et al Heart disease and stroke statistics—2014 update: a report from the American Heart Association. Circulation 2014;129:e28–e292 24352519 10.1161/01.cir.0000441139.02102.80 PMC5408159

[13] Konstantinides SV , Meyer G, Becattini C, Bueno H, Geersing GJ, Harjola VP et al 2019 ESC guidelines for the diagnosis and management of acute pulmonary embolism developed in collaboration with the European Respiratory Society (ERS): the task force for the diagnosis and management of acute pulmonary embolism of the European Society of Cardiology (ESC). Eur Respir J 2019;54:1901647 31473594 10.1183/13993003.01647-2019

[14] Aujesky D , Obrosky DS, Stone RA, Auble TE, Perrier A, Cornuz J et al Derivation and validation of a prognostic model for pulmonary embolism. Am J Respir Crit Care Med 2005;172:1041–1046 16020800 10.1164/rccm.200506-862OC PMC2718410

[15] Keeling WB , Leshnower BG, Lasajanak Y, Binongo J, Guyton RA, Halkos ME et al Midterm benefits of surgical pulmonary embolectomy for acute pulmonary embolus on right ventricular function. J Thorac Cardiovasc Surg 2016;152:872–878 26992603 10.1016/j.jtcvs.2015.11.042

[16] Pasrija C , Kronfli A, Rouse M, Raithel M, Bittle GJ, Pousatis S et al Outcomes after surgical pulmonary embolectomy for acute submassive and massive pulmonary embolism: a single-center experience. J Thorac Cardiovasc Surg 2018;155:1095–106.e2 29452460 10.1016/j.jtcvs.2017.10.139

[17] Neely RC , Byrne JG, Gosev I, Cohn LH, Javed Q, Rawn JD et al Surgical embolectomy for acute massive and submassive pulmonary embolism in a series of 115 patients. Ann Thorac Surg 2015;100:1245–1251; discussion 1251–1252 26165484 10.1016/j.athoracsur.2015.03.111

[18] Iaccarino A , Frati G, Schirone L, Saade W, Iovine E, D’Abramo M et al Surgical embolectomy for acute massive pulmonary embolism: state of the art. J Thorac Dis 2018;10:5154–5161 30233892 10.21037/jtd.2018.07.87 PMC6129933

[19] Chatterjee S , Chakraborty A, Weinberg I, Kadakia M, Wilensky RL, Sardar P et al Thrombolysis for pulmonary embolism and risk of all-cause mortality, major bleeding, and intracranial hemorrhage: a meta-analysis. JAMA 2014;311:2414–2421 24938564 10.1001/jama.2014.5990

[20] Marti C , John G, Konstantinides S, Combescure C, Sanchez O, Lankeit M et al Systemic thrombolytic therapy for acute pulmonary embolism: a systematic review and meta-analysis. Eur Heart J 2015;36:605–614 24917641 10.1093/eurheartj/ehu218 PMC4352209

[21] Hickey GL , Grant SW, Cosgriff R, Dimarakis I, Pagano D, Kappetein AP et al Clinical registries: governance, management, analysis and applications. Eur J Cardiothorac Surg 2013;44:605–614 23371972 10.1093/ejcts/ezt018

[22] Youden WJ . Index for rating diagnostic tests. Cancer 1950;3:32–35 15405679 10.1002/1097-0142(1950)3:1<32::aid-cncr2820030106>3.0.co;2-3

[23] Dohle K , Dohle DS, El Beyrouti H, Buschmann K, Emrich AL, Brendel L et al Short- and long-term outcomes for the surgical treatment of acute pulmonary embolism. Innov Surg Sci 2018;3:271–276 31579791 10.1515/iss-2018-0024 PMC6604590

[24] Lehnert P , Lange T, Møller CH, Olsen PS, Carlsen J. Acute pulmonary embolism in a national Danish cohort: increasing incidence and decreasing mortality. Thromb Haemost 2018;118:539–546 29536465 10.1160/TH17-08-0531

[25] Kilic A , Shah AS, Conte JV, Yuh DD. Nationwide outcomes of surgical embolectomy for acute pulmonary embolism. J Thorac Cardiovasc Surg 2013;145:373–377 22341655 10.1016/j.jtcvs.2012.01.066

[26] Percy ED , Shah R, Hirji S, Tartarini RJ, Yazdchi F, Harloff M et al National outcomes of surgical embolectomy for acute pulmonary embolism. Ann Thorac Surg 2020;110:441–447 32199827 10.1016/j.athoracsur.2020.02.024

[27] Alqahtani F , Munir MB, Aljohani S, Tarabishy A, Almustafa A, Alkhouli M. Surgical thrombectomy for pulmonary embolism: updated performance rates and outcomes. Tex Heart Inst J 2019;46:172–174 31708697 10.14503/THIJ-18-6751 PMC6827469

[28] Kon ZN , Pasrija C, Bittle GJ, Vemulapalli S, Grau-Sepulveda MV, Matsouaka R et al The incidence and outcomes of surgical pulmonary embolectomy in North America. Ann Thorac Surg 2019;107:1401–1408 30476479 10.1016/j.athoracsur.2018.10.035

[29] Kadner A , Schmidli J, Schonhoff F, Krahenbuhl E, Immer F, Carrel T et al Excellent outcome after surgical treatment of massive pulmonary embolism in critically ill patients. J Thorac Cardiovasc Surg 2008;136:448–451 18692656 10.1016/j.jtcvs.2007.11.021

[30] Vohra HA , Whistance RN, Mattam K, Kaarne M, Haw MP, Barlow CW et al Early and late clinical outcomes of pulmonary embolectomy for acute massive pulmonary embolism. Ann Thorac Surg 2010;90:1747–1752 21095299 10.1016/j.athoracsur.2010.08.002

[31] Mkalaluh S , Szczechowicz M, Karck M, Szabo G. Twenty-year results of surgical pulmonary thromboembolectomy in acute pulmonary embolism. Scand Cardiovasc J 2019;53:98–103 30919668 10.1080/14017431.2019.1600013

[32] Lee T , Itagaki S, Chiang YP, Egorova NN, Adams DH, Chikwe J. Survival and recurrence after acute pulmonary embolism treated with pulmonary embolectomy or thrombolysis in New York State, 1999 to 2013. J Thorac Cardiovasc Surg 2018;155:1084–90.e12 28942971 10.1016/j.jtcvs.2017.07.074

[33] Choi JH , O'Malley TJ, Maynes EJ, Weber MP, D'Antonio ND, Mellado M et al Surgical pulmonary embolectomy outcomes for acute pulmonary embolism. Ann Thorac Surg 2020;110:1072–1080 32151576 10.1016/j.athoracsur.2020.01.075

[34] Minakawa M , Fukuda I, Miyata H, Motomura N, Takamoto S, Taniguchi S et al Outcomes of pulmonary embolectomy for acute pulmonary embolism. Circ J 2018;82:2184–2190 29952349 10.1253/circj.CJ-18-0371

[35] Kline JA , Steuerwald MT, Marchick MR, Hernandez-Nino J, Rose GA. Prospective evaluation of right ventricular function and functional status 6 months after acute submassive pulmonary embolism: frequency of persistent or subsequent elevation in estimated pulmonary artery pressure. Chest 2009;136:1202–1210 19542256 10.1378/chest.08-2988 PMC2818852

[36] Ahmed P , Khan AA, Smith A, Pagala M, Abrol S, Cunningham JN Jr et al Expedient pulmonary embolectomy for acute pulmonary embolism: improved outcomes. Interact Cardiovasc Thorac Surg 2008;7:591–594 18469011 10.1510/icvts.2008.176735

[37] Ius F , Hoeper MM, Fegbeutel C, Kuhn C, Olsson K, Koigeldiyev N et al Extracorporeal membrane oxygenation and surgical embolectomy for high-risk pulmonary embolism. Eur Respir J 2019;53:1801773 30728206 10.1183/13993003.01773-2018

[38] Keeling WB , Sundt T, Leacche M, Okita Y, Binongo J, Lasajanak Y et al Outcomes after surgical pulmonary embolectomy for acute pulmonary embolus: a multi-institutional study. Ann Thorac Surg 2016;102:1498–1502 27373187 10.1016/j.athoracsur.2016.05.004

